# Neuroinflammation in the anterior cingulate cortex: the potential supraspinal mechanism underlying the mirror-image pain following motor fiber injury

**DOI:** 10.1186/s12974-022-02525-8

**Published:** 2022-06-20

**Authors:** Qiao-Yun Li, Shao-Xia Chen, Jin-Yu Liu, Pei-Wen Yao, Yi-Wen Duan, Yong-Yong Li, Ying Zang

**Affiliations:** 1 Pain Research Center and Department of Physiology, Zhongshan Medical School of Sun Yat-Sen University, Guangdong Provincial Key Laboratory of Brain Function and Disease, 74 Zhongshan Rd. 2, Guangzhou, 510080 People’s Republic of China; 2 Department of Anesthesiology, Sun Yat-Sen University Cancer Center, State Key Laboratory of Oncology in South China, Collaborative Innovation Center for Cancer Medicine, 651 Dongfeng Road East, Guangzhou, 510060 People’s Republic of China

**Keywords:** Neuropathic pain, Mirror-image pain, Anterior cingulate cortex, Spinal cord, Chemokine

## Abstract

**Background:**

Peripheral nerve inflammation or lesion can affect contralateral healthy structures, and thus result in mirror-image pain. Supraspinal structures play important roles in the occurrence of mirror pain. The anterior cingulate cortex (ACC) is a first-order cortical region that responds to painful stimuli. In the present study, we systematically investigate and compare the neuroimmune changes in the bilateral ACC region using unilateral- (spared nerve injury, SNI) and mirror-(L5 ventral root transection, L5-VRT) pain models, aiming to explore the potential supraspinal neuroimmune mechanism underlying the mirror-image pain.

**Methods:**

The up-and-down method with von Frey hairs was used to measure the mechanical allodynia. Viral injections for the designer receptors exclusively activated by designer drugs (DREADD) were used to modulate ACC glutamatergic neurons. Immunohistochemistry, immunofluorescence, western blotting, protein microarray were used to detect the regulation of inflammatory signaling.

**Results:**

Increased expressions of tumor necrosis factor alpha (TNF-α), interleukin-6 (IL-6) and chemokine CX3CL1 in ACC induced by unilateral nerve injury were observed on the contralateral side in the SNI group but on the bilateral side in the L5-VRT group, representing a stronger immune response to L5-VRT surgery. In remote ACC, both SNI and L5-VRT induced robust bilateral increase in the protein level of Nav1.6 (*SCN8A*), a major voltage-gated sodium channel (VGSC) that regulates neuronal activity in the mammalian nervous system. However, the L5-VRT-induced Nav1.6 response occurred at PO 3d, earlier than the SNI-induced one, 7 days after surgery. Modulating ACC glutamatergic neurons via DREADD-Gq or DREADD-Gi greatly changed the ACC CX3CL1 levels and the mechanical paw withdrawal threshold. Neutralization of endogenous ACC CX3CL1 by contralateral anti-CX3CL1 antibody attenuated the induction and the maintenance of mechanical allodynia and eliminated the upregulation of CX3CL1, TNF-α and Nav1.6 protein levels in ACC induced by SNI. Furthermore, contralateral ACC anti-CX3CL1 also inhibited the expression of ipsilateral spinal c-Fos, Iba1, CD11b, TNF-α and IL-6.

**Conclusions:**

The descending facilitation function mediated by CX3CL1 and its downstream cascade may play a pivotal role, leading to enhanced pain sensitization and even mirror-image pain. Strategies that target chemokine-mediated ACC hyperexcitability may lead to novel therapies for the treatment of neuropathic pain.

**Supplementary Information:**

The online version contains supplementary material available at 10.1186/s12974-022-02525-8.

## Background

Chronic neuropathic pain (NP) results from multiple etiological factors that initiate diverse mechanisms and express both within, and across different disease states [1]. It is now clear that peripheral nerve inflammation or lesion can affect contralateral healthy structures, and thus results in mirror-image pain [2–10], a clinical pain-associated phenomenon [11–14]. Although the contralateral/mirror pain is commonly reported, it is hard to achieve satisfying therapeutic effect. Understanding the signaling mechanisms underlying mirror-image pain may provide novel therapeutic targets for NP.

Cytokines have been suggested to cause mirror-image pain. Most of previous studies focus on the spinal cord, indicating a key role of activated astrocytes, microglia and proinflammatory cytokines in mirror pain [3, 10, 15–19]. In fact, unilateral damage to peripheral nerves or spinal roots produces many breakdown products and development of an aseptic inflammatory reaction. Released cytokines are believed to be transported via blood or cerebrospinal fluid (CSF) to the contralateral site affecting dorsal root ganglia (DRG) or peripheral nerves [7–9, 15, 20–22]. Thus, the peripheral nervous system is involved in the development of mirror-image pain.

Furthermore, others suggested that supraspinal structures may play important roles in the occurrence of mirror pain. For instance, the opioid receptor signaling in the thalamic submedius and ventromedial nuclei are inhibitory of mirror or contralateral pain whereas the mediodorsal (MD) nucleus, a key relay for spinal nociceptive inputs [23], facilitates the mirror or contralateral pain [6, 24, 25]. The anterior cingulate cortex (ACC) is a first-order cortical region that responds to painful stimuli [26]. Others and our recent work show a role of ACC in pain processing and pain-related aversion [26–34]. The thalamic MD nucleus with connections to the ACC, a projection that relays nociceptive input for central processing [35–37], participates in emotional and discrimination aspects of pain with a corresponding nociceptive facilitation [24, 28, 31, 38]. However, the supraspinal neuroimmune mechanism underlying contralateral pain [39] has not been extensively revealed. In our recent work, we used the spared (sciatic) nerve injury (SNI) model that displays unilateral mechanical allodynia and thermal hyperalgesia [40] and found that increased tumor necrosis factor alpha (TNF-α) in contralateral ACC contributes to pain aversiveness and pain maintenance [33].

Ectopic discharges from uninjured but not injured afferents are important for the development of neuropathic pain [41]. Selective injury to the motor fibers, leaving the sensory fibers intact, by L5 ventral root transection (L5-VRT) can reliably induce mirror-image pain [2, 9, 10, 21, 42–44]. In the present study, we systematically investigate and compare the spatiotemporal expression variations of chemokine CX3CL1, proinflammatory cytokines (TNF-α/interleukin-6) as well as voltage-gated Nav1.6 sodium channel in bilateral ACC region of SNI and L5-VRT models, and discuss the possible mechanism underlying CX3CL1’s involvement in ACC's descending facilitation of spinal cord. This study aims to explore the potential supraspinal neuroimmune mechanism underlying the mirror-image pain.

## Methods

### Animals

Adult male Sprague-Dawley rats (160–200 g) and C57BL/6 mice (20–26 g) were obtained from the Institute of Experimental Animals, Sun Yat-Sen University, China (License number SCXK (yue) 2008-0002). Animals were housed in separate cages with controlled humidity (50–60%), temperature (24 ℃), and 12-h light/dark cycle (06:00–18:00 h). Food and water were available ad libitum.

### Spared nerve injury (SNI) of sciatic nerve

SNI surgery was performed as described previously [40]. Briefly, rats or mice were anaesthetized intraperitoneally (i.p.) with 0.4% sodium pentobarbital (40 mg/kg, Sigma-Aldrich). The skin on the lateral surface of the left thigh was incised and then sectioned to expose the sciatic nerve and its three terminal branches: sural, common peroneal and tibial nerves. The common peroneal and tibial nerves were ligated and sectioned (removal of a 2 mm length), leaving the sural nerve intact. In the sham-operated group, an identical operation was performed to expose the nerves without being injured.

### L5 ventral root transection (L5-VRT)

L5-VRT surgery was performed as described by Li et al. [2]. Animals were anesthetized similarly to SNI model. Briefly, a left L5 hemi-laminectomy was performed to expose the left L5 nerve root. The ventral root was pulled out with fine forceps. The dissection was then performed 2–3 mm proximal to the DRG, and a small portion (2 mm) of the root was removed. In the sham group, the ventral root was only exposed [2].

### Mechanical allodynia test

The up-and-down method with von Frey hairs was used to measure the mechanical allodynia in the hind paws of rats and mice [45]. Briefly, animals were placed in separate plexiglass chambers positioned on a mesh table. The allodynia test was performed after 15 min of habituation. Starting with a dose of 2.04 g (rat) and 0.40 g (mouse), von Frey hairs of logarithmically incremental stiffness (rat: 0.41, 0.70, 1.20, 2.04, 3.63, 5.50, 8.51, 15.14 g; mouse: 0.04, 0.07, 0.16, 0.40, 0.60, 1.0, 1.4, 2.0 g) were applied bilaterally to hindpaws. Fifty percent paw withdrawal thresholds were recorded and the response to mechanical stimuli was evaluated.

### Immunohistochemistry and immunofluorescence

As described previously [33], animals were anesthetized and perfused through the ascending aorta with 0.9% saline followed by 4% paraformaldehyde in 0.1 M phosphate buffer (PB). Following perfusion brains were removed and postfixed for 5 h, then dehydrated in 30% sucrose for 5 days. After that, brain tissue containing ACC (bregma + 3.0 ~  + 1.7 mm) was coronally sliced (25 µm thickness) with a freezing microtome (LEICA CM3050S, Germany).

For immunohistochemistry, sections were first blocked with 5% donkey serum for 1 h at room temperature, then incubated with mouse anti-c-Fos antibody (1:200, Millipore, Germany) or rabbit anti-TNF-α antibody (1:100; Bioworld, USA) for overnight at 4 °C. After three PBS washes, 10 min/each, the sections were incubated with a Cy3-conjugated goat anti-mouse (or anti-rabbit) secondary antibody (1:400; Jackson Immuno Research, USA) for 1 h at room temperature, and then washed with PBS.

For double immunofluorescence staining, the brain sections were incubated with a mixture of sodium channel voltage-gated type VIII alpha subunit antibody (anti-Nav1.6 antibody; 1:100; Alomone, Israel) plus either anti-TNF-α antibody (1:100; Bioworld, USA), anti-IL-6 antibody (1:200; CST, USA), monoclonal neuronal-specific nuclear protein (mouse anti-NeuN; neuronal marker, 1:200; Chemicon, USA), glial fibrillary acidic protein (mouse anti-GFAP, astrocyte marker, 1:400; CST, USA) or goat anti-Iba1 (microglia marker, 1:200; Abcam, UK) for overnight at 4 °C. Double-immunofluorescence staining of anti-IL-6 antibody with anti-NeuN, anti-GFAP or anti-Iba1 and of fractalkine chemokine domain antibody (anti-CX3CL1 antibody; 1:100; R&D systems, USA) with anti-NeuN, anti-GFAP or anti-Iba1 in the brain sections were also made. After three rinses with PBS, 10 min/each, sections were incubated with fluorescein isothiocyanate (FITC)- and Cy3-conjugated secondary antibodies (1:400; Jackson Immuno Research, USA) for 1 h at room temperature, followed by PBS washes.

For imaging, sections were mounted on gelatin-coated slides and air-dried. Images were obtained using a fluorescence microscope attached to a CCD spot camera (LEICA DFC350FX/DMIRB, Germany) and processed with LEICA IM50 software (Germany). To verify specificity of the immunostaining and primary antibodies, negative control sections were processed in parallel without primary antibodies (data not shown).

### Western blotting

**Fig. 1:**
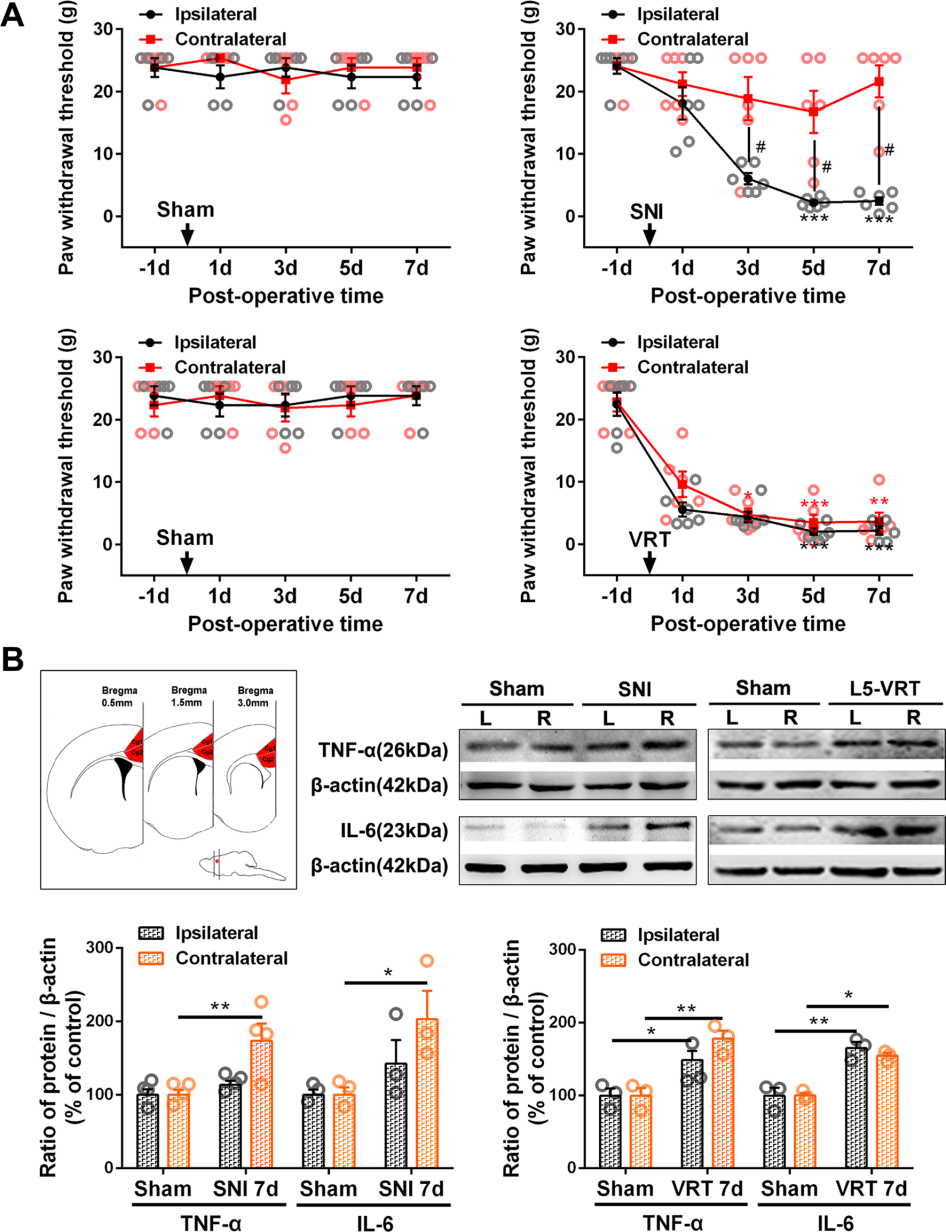
Comparison of L5-VRT and SNI-induced mechanical allodynia and expression of TNF-α and IL-6 in bilateral ACC. **A** Changes in bilateral mechanical paw withdrawal thresholds of SNI and L5-VRT operated rats. Significant differences are observed in threshold for ipsilateral paw in rats with SNI and bilateral paws in rats with L5-VRT on day 3, 5 and 7. *n* = 6 rats in each group. Statistical significance is determined by Dunn's multiple comparisons test (*p < 0.05; **p < 0.01; ***p < 0.001 versus PO-1d) or multiple t tests (#*p* < 0.05 versus contralateral side). **B** L5-VRT and SNI-induced expression of TNF-α and IL-6 proteins in bilateral ACC. Upper left, the tissue of Cg1 and Cg2 (red region) was used for western blotting. Sections were in the coronal plane, number in mm anterior to Bregma indicated in this and subsequent figures. Representative western blotting of TNF-α and IL-6 protein levels in bilateral ACC are shown on the upper right. The quantification results are shown below. Significant differences are observed on the contralateral side in the SNI group but on the bilateral side in the L5-VRT group 7d after nerve injury. *p < 0.05, **p < 0.01 versus sham group (one-way ANOVA)

Rats were euthanized at designated time points and tissue samples of ACC were quickly dissected from brain slices (bregma + 3.0 ~  + 0.5 mm) using an anatomical microscope (red area in Fig. 1B). Tissue samples were centrifuged at 12,000 rpm for 20 min at 4 ℃ and proteins were quantified. Proteins were separated by gel electrophoresis (SDS-PAGE) and electro-transferred to a PVDF membrane (Millipore). After blocking with 5% nonfat milk (containing Tris–phosphate buffer, 0.05% Tween 20) for 1 h at room temperature, the membrane was incubated overnight at 4 ℃ with mouse anti-c-Fos antibody (1:200; Millipore, Germany), polyclonal rabbit TNF-α antibody (1:1000; Bioworld, USA), anti-IL-6 antibody (1:1000; Abcam, UK), anti-CX3CL1 antibody (1:1000; Abcam, UK), anti-Nav1.6 antibody (1:200; Alomone, Israel), anti-CD11b antibody (1:1000; Bioss, China) or anti-Iba1 (1:1000; Abcam, UK). β-actin was used (1:1000, Boster, Germany) as a loading control. The blots were washed three times with TBS-T for 10 min., and then incubated with HRP conjugated donkey anti-mouse, anti-goat or anti-rabbit secondary antibodies (1:10,000; Abcam, UK). The target protein bands were detected using enhanced chemiluminescence (Bio-Rad) and imaged using a Tanon-5200 Chemiluminescent Imaging System (Tanon Science and Technology). The protein level was quantified by densitometry using an imaging analysis system (KONTRON IBAS 2.0, Germany) and expressed relative to the level of β-actin.

### Viral injections for the designer receptors exclusively activated by designer drugs (DREADD)

In our previous work [33], injection of the hM4Di-mCherry virus into SNI rats and mice significantly increases SNI-induced paw withdrawal threshold, but compared with mice, the decreased threshold in SNI rats is not completely reversed to the base line following administration of CNO. Considering the viral transfection efficiency, in this work, we applied chemogenetic viruses to mice.

As described previously [33], mice were deeply anesthetized (1–2% isoflurane) and then mounted in a stereotaxic frame with nonpuncturing ear bars. To selectively express hM4Di (Gi-coupled human M4 muscarinic receptor) or hM3Dq (Gq-coupled human M3 muscarinic receptor) in ACC excitatory glutamatergic neurons, the pAOV-CaMKIIa-hM4D(Gi)-mCherry-3Flag (hM4Di-mCherry) or pAAV-CaMKIIa-hM3D(Gq)-mCherry (hM3Dq-mCherry) were injected into the contralateral (right) ACC of mice with or without SNI surgery, respectively. In parallel, all control mice received right injections of pAOV-CaMKIIa-mCherry-3Flag or pAAV-CaMKIIa-mCherry (mCherry). Viral particles (approximate titer 1.0E + 12 GC/ml, Obio Technology Shanghai Corp., Ltd.) were administrated using a nanoinjector with injection micropipette (Nanoject II Auto-Nanoliter Injector, DRUMMOND, USA) at the following coordinates: anteroposterior (AP) + 1.0 mm, mediolateral (ML) 0.2 mm, dorsoventral (DV) − 1.2 mm. A total volume of 500 nl was injected at the speed of 23 nl per second. After injection, incisions were stitched and mice were individually housed for 2 weeks before behavioral tests. At designated time points animals received i.p. injections of the ligand clozapine-*N*-oxide (CNO, Sigma-Aldrich) at a dose of 10 μl/g body weight. CNO working solution was first dissolved in dimethylsulfoxide (DMSO) and then diluted to a final concentration (5.0 mg/kg) with saline. The final concentration of DMSO was 0.5%.

### Chemokine measurements with use of protein microarray

Rats were euthanized at designated time points and tissue samples of ACC were quickly dissected from brain slices (bregma + 3.0 ~  + 0.5 mm) using an anatomical microscope. Tissue was excised and homogenized in PBS with protease inhibitors. After homogenization, Triton X-100 was added to a final concentration of 1%. Tissue samples were centrifuged at 10,000 rpm for 5 min at 4 °C and proteins were quantified. Samples were assay immediately or stored in aliquots at − 70 ℃. Duplicate levels of 20 secreted proteins were determined using the Proteome Profiler mouse chemokine array kit according to manufacturer’s instructions (R&D systems, Proteome Profiler Rat XL Cytokine Array. Catalog Number: ARY030). Blots were imaged using enhanced chemiluminescence (Bio-Rad) and imaged using a Tanon-5200 Chemiluminescent Imaging System (Tanon Science and Technology). The band intensity of each blot was analyzed by densitometry using an imaging analysis system (KONTRON IBAS 2.0, Germany) and expressed relative to negative control spots or a clear area of the array.

### Intra-ACC drug microinjection

As described previously [33], stereotaxic surgery was performed on anesthetized rats (10% chloralhydrate, 0.4 g/kg, i.p.) according to the rat brain atlas animals. A stainless-steel guide cannula with a stainless-steel stylet plug was inserted into the ACC on the opposite side of the operation and secured with dental acrylic cement. The stereotaxic coordinate for ACC injection site from bregma was as follows: AP + 2.0 mm, ML 0.5 mm, DV-2.5 mm. After a week of recovery from catheterization, anti-rat CX3CL1 antibody (AF-510-NA, R&D systems, Inc.) or normal goat IgG control (AB-108-C, R&D systems, Inc.) were injected into the ACC at a dose of 10 μg/ml (10 μl, R&D) over a 5 min period, 30 min before or 7 d after SNI.

### Statistical analysis

For immunohistochemistry, a density threshold above background level was first established to identify positively stained structures. For each animal, five slices were extracted from a series of consecutive ACC slices (four slice intervals) for statistical analysis. The immunofluorescence intensity per slice in the same Cg1 region of ACC (300 × 300 pixels) was measured and the mean ± SEM across animals was determined.

Changes in values over different groups were tested using one-way ANOVA followed by Dunnett's multiple comparisons test or using two-way ANOVA followed by Sidak's multiple comparisons test. For behavioral testing data, nonparametric two-way ANOVA followed by Friedman test was employed. In all cases, *p* < 0.05 was considered statistically significant.

## Results

### Motor fiber injury by L5-VRT induces stronger immune activation in bilateral ACC compared to SNI model

Consistent with earlier findings [2, 40], unilateral L5-VRT induced significant decrease in bilateral paw withdrawal thresholds (*p* < 0.05) at postoperative (PO) day 3 till day 7, while SNI rats only showed mechanical allodynia on the injured side (Fig. 1A). Levels of proinflammatory cytokines TNF-α and IL-6 were investigated by western blotting to compare variations in immune activation between the ACCs of SNI and L5-VRT models. Elevated expressions of TNF-α and IL-6 in ACC induced by unilateral nerve injury were observed on the contralateral side in the SNI group but on the bilateral side in the L5-VRT group 7 days (7d) after nerve injury (Fig. 1B). Sham operation had no significant effects on paw withdrawal thresholds, TNF-α or IL-6 levels.

**Fig. 2:**
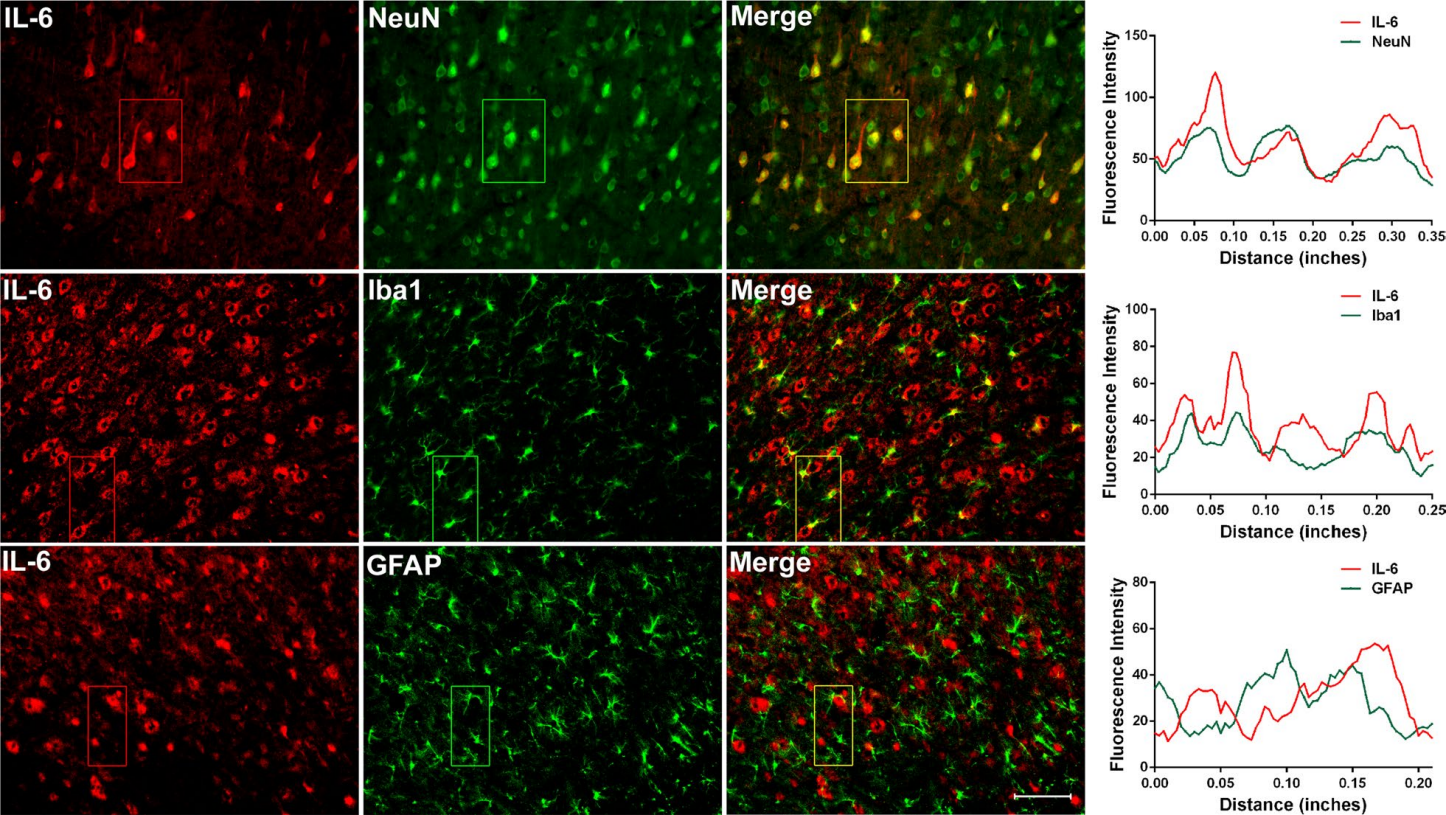
Nerve injury-induced IL-6 is expressed in ACC neurons and microglia cells. Double-immunofluorescence staining showing the co-localization of IL-6-IR (red) with NeuN (neuronal marker, green, top) and Iba1 (microglia marker, green, medial), but not GFAP (astrocyte marker, green, below) 7d post-SNI. Scale bar = 50 μm. The fluorescence intensity curves for red and green from boxed areas are shown on the right side of each group

Dual immunolabeling studies were performed to identify the cells responsible for abnormal levels of cytokines in ACC. Previous study indicated that TNF-α colocalizes with NeuN (neuronal marker), but not GFAP (astrocyte marker) or Iba1 (microglial marker) 7d after SNI [33]. An important finding in this study is that IL-6 colocalizes with Iba1 and not only with NeuN (Fig. 2). Having in mind that SNI induces the activation of amoeboid shaped microglia in the ACC [33], this finding suggests that the immune response secondary to the activation of microglia may also be involved in the processing of pain information in the ACC.

**Fig. 3:**
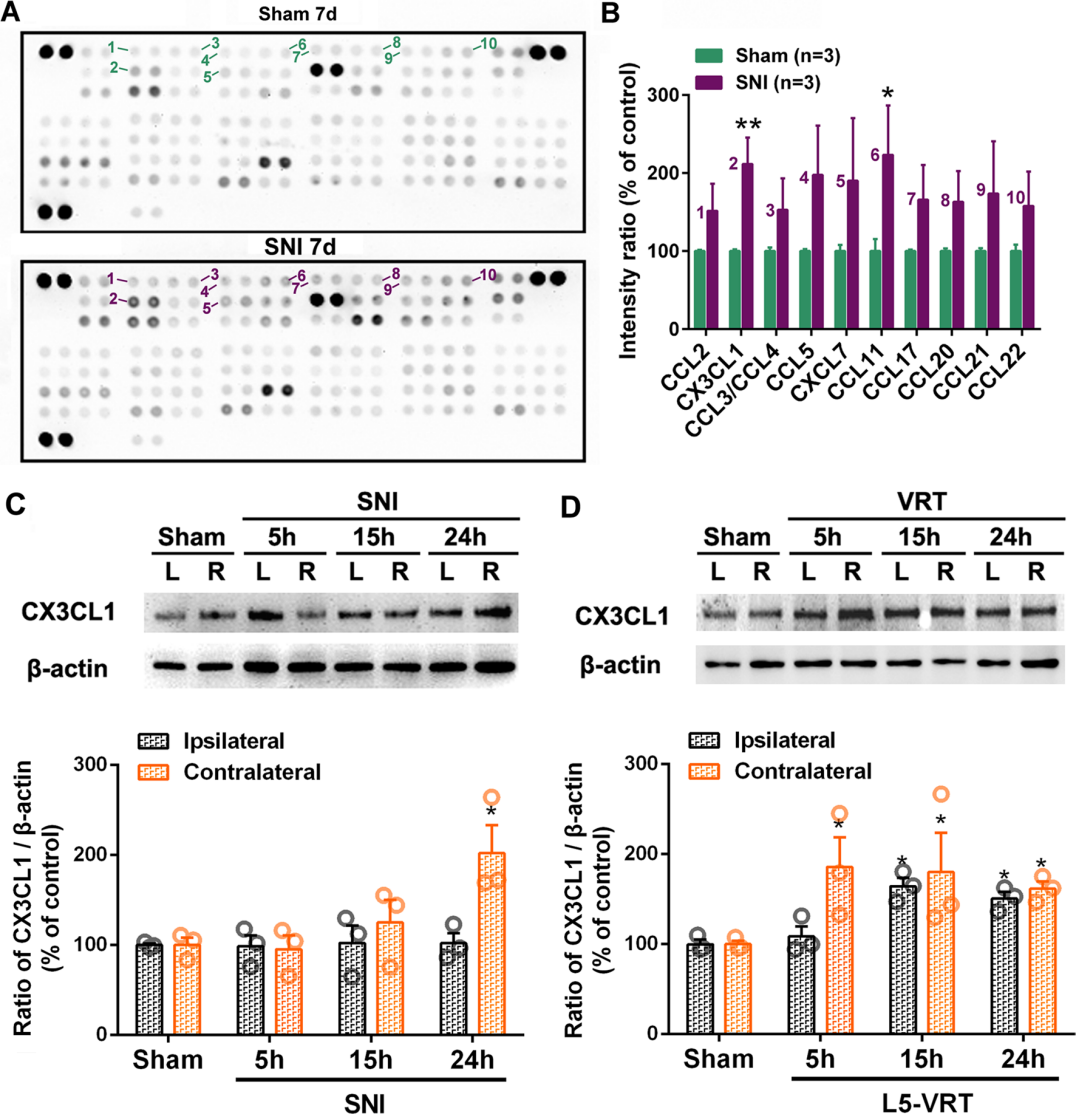
Comparison of L5-VRT and SNI-induced expression of CX3CL1 in bilateral ACC. Representative protein microarray results (**A**) and the quantification analysis (**B**) showing that SNI triggered upregulations of chemokines CX3CL1 and CCL11 in contralateral ACC.*p < 0.05, **p < 0.01 versus sham group (*n* = 3 rats/group, one-way ANOVA). **C**, **D** Representative western blotting of CX3CL1 protein levels in bilateral ACC following SNI (**C**) and L5-VRT (**D**) are shown on the top and the quantification results are shown below. Significant differences are observed on the contralateral side in the SNI group but on the bilateral side in the L5-VRT group. *p < 0.05 versus sham group (*n* = 3 rats/group, one-way ANOVA)

In addition to proinflammatory cytokines, chemokines are also important molecules that mediate neuroinflammation and play an important role in the formation and maintenance of neuropathic pain [46]. Protein microarray indicated that SNI induced significant increases in the levels of chemokines CX3CL1 and CCL11 in contralateral ACC (Fig. 3A, B). Due to the uncertainty of the role of CCL11 in chronic pain [47–49], this study focuses on determining the expression of CX3CL1 in ACC in animals with or without nerve injury. To compare the early neuroinflammatory changes between SNI and L5-VRT, the protein level of CX3CL1 within 24 h following nerve injury was detected. Western blot analysis showed that SNI-induced CX3CL1 levels significantly increase in contralateral but not ipsilateral ACC at PO 24 h (*p* < 0.05, Fig. 3C), while VRT-induced increase in CX3CL1 was first observed in contralateral ACC at PO 5 h and bilateral at PO 15 and 24 h (*p* < 0.05, Fig. 3D), representing a stronger immune response to L5-VRT surgery. Our previous work has shown that the decreased paw withdrawal threshold induced by L5-VRT are detected ipsilaterally at 15 h and contralaterally at 24 h, lasting at least for 4 weeks. In contrast, no changes in paw withdrawal thresholds were detected in sham-operated rats [50]. The abnormal expression of CX3CL1 in ACC precedes the induction of mechanical allodynia.

**Fig. 4:**
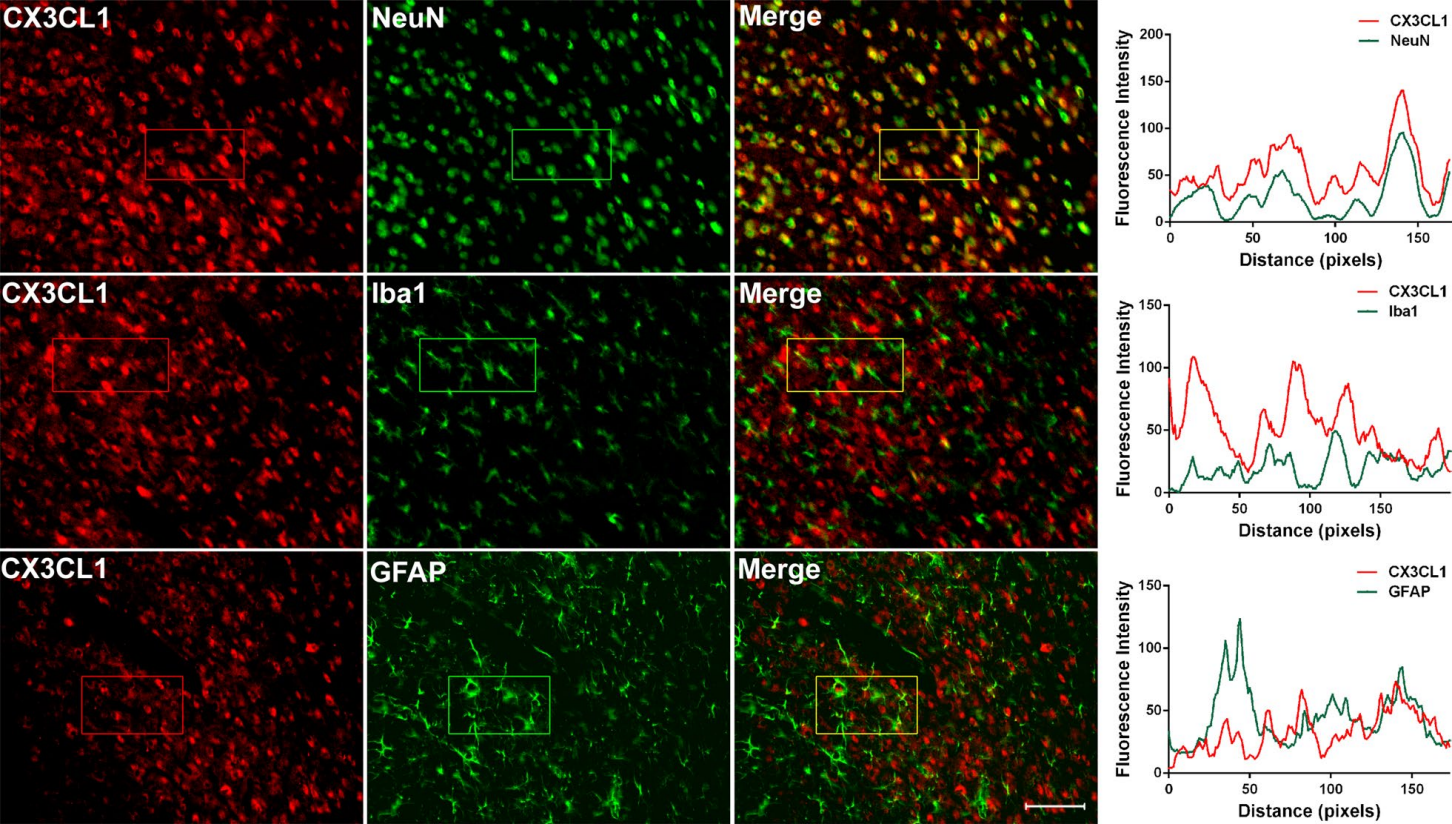
Nerve injury-induced CX3CL1 is expressed in ACC neurons. Double-immunofluorescence staining showing the co-localization of CX3CL1-IR (red) with NeuN (green, top), but not Iba1 (green, medial) and GFAP (green, below) 7d post-SNI. Scale bar = 50 μm. The fluorescence intensity curves for red and green from boxed areas are shown on the right side of each group

Dual immunolabeling in ACC showed that CX3CL1 co-localized mainly with NeuN, but not Iba1 or GFAP following SNI (Fig. 4), which was like the cellular localization in spinal cord [46].

### The effect of modulating the excitability of ACC glutamatergic neurons on CX3CL1 expression

To modulate the excitability of ACC, the chemogenetic method of DREADD was used to selectively express hM4Di or hM3Dq in ACC excitatory glutamatergic neurons. The co-labeling of the immunoreactivity (IR) of mCherry (virus marker) and CaMKII was analyzed to reflect the transfection specificity of the virus (Additional file 1: Fig. S1). The co-localization numbers of mCherry-IR, hM3Dq-mCherry-IR, hM4Di-mCherry-IR and CaMKII-IR neurons accounted for 90.89 ± 1.62%, 86.11 ± 4.97%, 78.87 ± 3.24% of the total number of transfected neurons, respectively, showing a good specificity of viral transfection.

Our previous work has shown that SNI raises the expression of c-Fos, a widely used neuronal activity marker, especially in contralateral ACC at early (PO 1 h and 1d) and late (PO 7and 10d) time points [33]. To further determine the regulation of ACC neuronal activity by DREADD-Gq or Gi, the quantity difference of co-labeled mCherry and c-Fos in different virus transfection groups was analyzed. As shown in Additional file 2: Fig. S2, compared with the mCherry control group, an increased co-labeled percentage of hM3Dq-mCherry-IR (red) and c-Fos-IR (green) and a decreased percentage of hM4Di-mCherry-IR (red) and c-Fos-IR (green) co-localized (yellow) in all transfected neurons (red) following CNO (5.0 mg/kg, i.p.) were observed, indicating that ACC activity could be effectively regulated by DREADD-Gq or Gi.

**Fig. 5:**
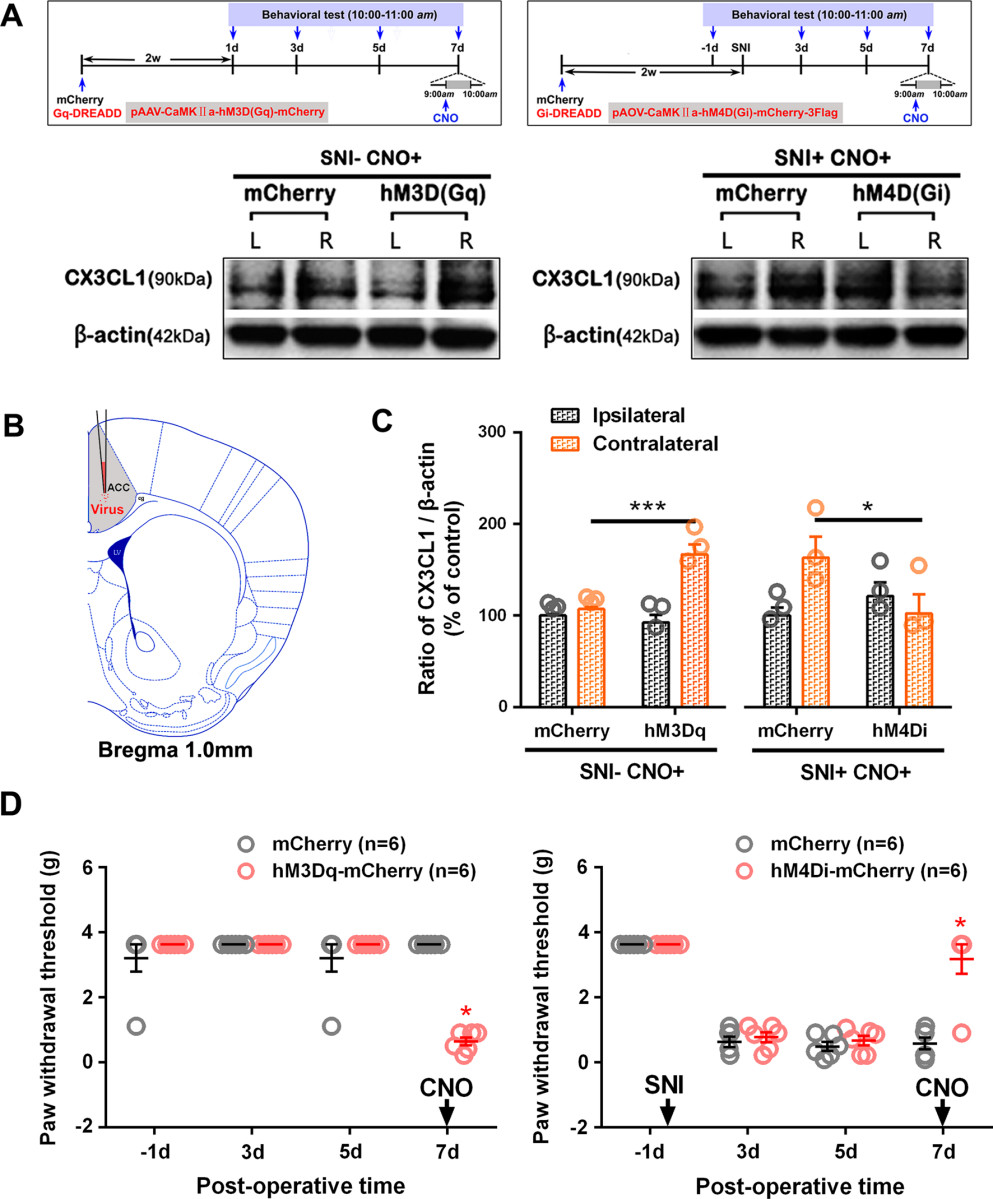
Effects of DREADD-hM3Dq and hM4Di on ACC CX3CL1 protein levels and the mechanical paw withdrawal thresholds. **A** Behavioral test paradigms are shown on the top and representative western blotting for bilateral ACC CX3CL1 in the mice injected with DREADD-Gq or DREADD-Gi are shown below. **B** The injection site of virus carrying hM3Dq-, hM4Di-mCherry or mCherry in contralateral ACC. **C** Quantification of bilateral CX3CL1 in the mice injected with DREADD-Gq and DREADD-Gi, respectively. *p < 0.05, ***p < 0.001 versus mCherry control groups (*n* = 3 mice/group, two-way ANOVA). **D** Compared to the mCherry control mice, DREADD-hM3Dq induced significant decrease of threshold in ipsilateral hind paws of normal mice (left); DREADD-hM4Di reversed the SNI-induced decrease of ipsilateral threshold (right). *p < 0.05 versus mCherry control groups (*n* = 6 mice/group, multiple t tests)

To verify whether the increased expression of CX3CL1 is a result of ACC hyperexcitation, the ACC glutamatergic neurons were inhibited in SNI mice by hM4Di-mCherry or excited by hM3Dq-mCherry in normal mice and the effect on CX3CL1 expression in ACC was determined. As shown in Fig. 5, in normal mice transfected with hM3Dq, the paw withdrawal thresholds decreased significantly (*p* < 0.05, Fig. 5D) 1 h after application of synthetic ligand CNO (5.0 mg/kg, i.p.), and the protein levels of CX3CL1 in the right ACC were upregulated dramatically (*p* < 0.001, Fig. 5A, C), suggesting that the induction of ACC hyperexcitation promotes the expression of CX3CL1. Furthermore, to examine the effect of inhibiting the activity of ACC glutamatergic neurons on CX3CL1 expression, SNI surgery was performed 2 weeks after hM4Di-mCherry virus injection and CNO was administrated at PO 7d. The inhibitory effects on the SNI-induced mechanical allodynia was determined (Fig. 5D) similarly to previous work [33]. Compared with the mCherry control group, the SNI-induced CX3CL1 overexpression in hM4Di group was greatly decreased (*p* < 0.05, Fig. 5A, C).

### Difference in expression of Nav1.6 protein in ACC neurons induced by SNI and L5-VRT

**Fig. 6:**
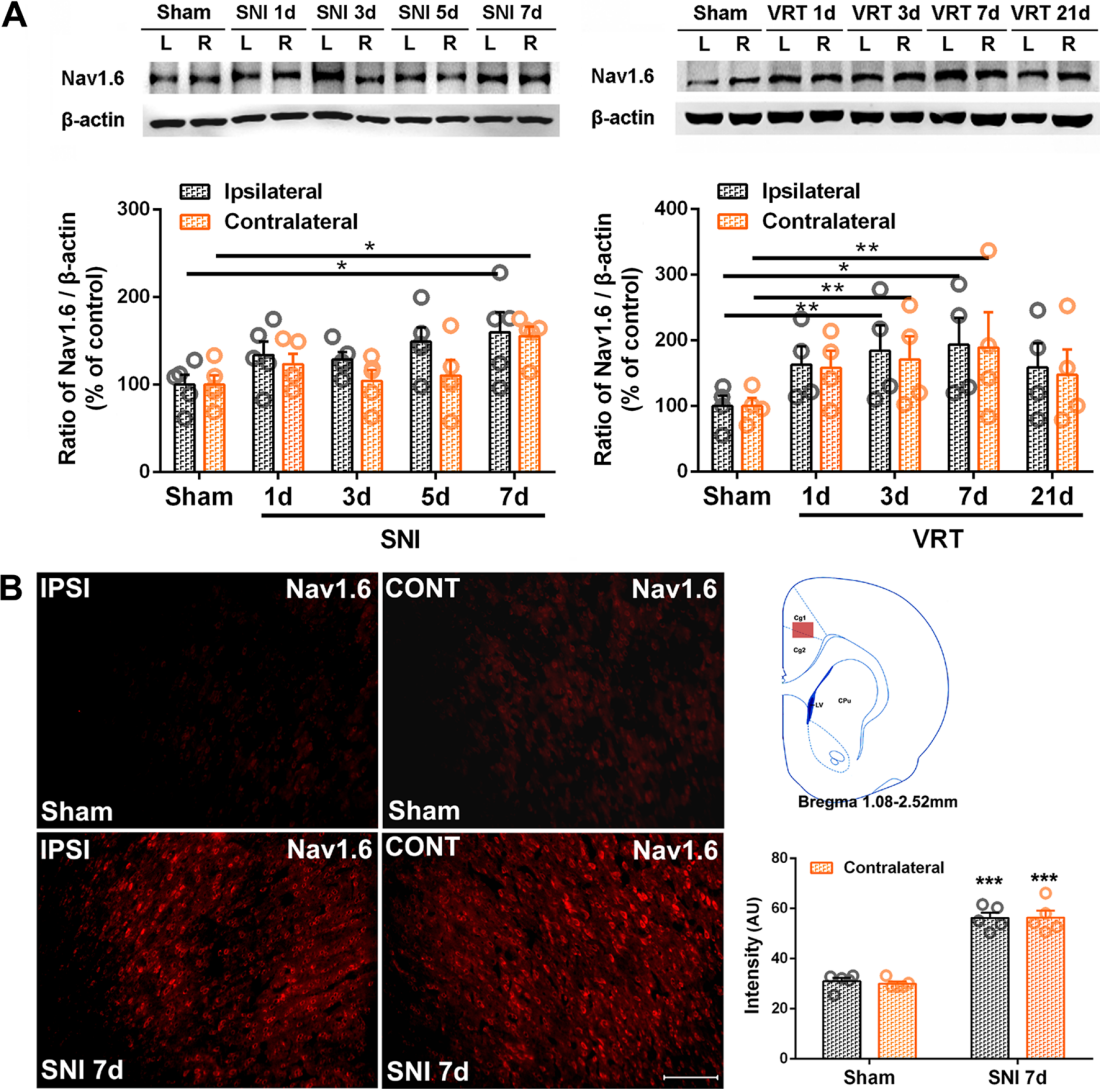
Analysis of the difference in protein levels of Nav1.6 in ACC induced by SNI and L5-VRT. **A** Representative western blotting of Nav1.6 protein levels in bilateral ACC following SNI and L5-VRT are shown on the top and the quantification results are shown below. *p < 0.05, **p < 0.01 versus sham group (one-way ANOVA). **B** The expression of Nav1.6-positive cells in bilateral ACC 7d post-SNI surgery. Representative data obtained from sham and SNI rats are shown. Scale bar = 100 μm. Detection area by red solid rectangular box is shown on the upper right and mean fluorescence intensity is shown on the lower right. ***p < 0.001 versus sham group (one-way ANOVA)

Nav1.6 (*SCN8A*) is a major voltage-gated sodium channel (VGSC) in the mammalian nervous system, where it regulates neuronal activity at the axon initial segment [51, 52] and is involved in the production and maintenance of pathological neuronal excitability in the peripheral nerves [53, 54], spinal cord [55] and supraspinal neurons [56]. Here, the changes in Nav1.6 protein levels over time was evaluated in SNI and L5-VRT induced ACC. Analysis by western blotting indicate that both SNI and L5-VRT induce robust bilateral increase in Nav1.6 protein levels in remote ACC. But the L5-VRT-induced Nav1.6 response occurred on the third day post-operation (PO 3d) (*p* < 0.01), earlier than that by SNI where it started at PO 7d (*p* < 0.05, Fig. 6A). The immunofluorescence labeling of Nav1.6 in ACC following SNI further confirmed its bilateral enhancement at PO 7d (*p* < 0.001, Fig. 6B).

**Fig. 7:**
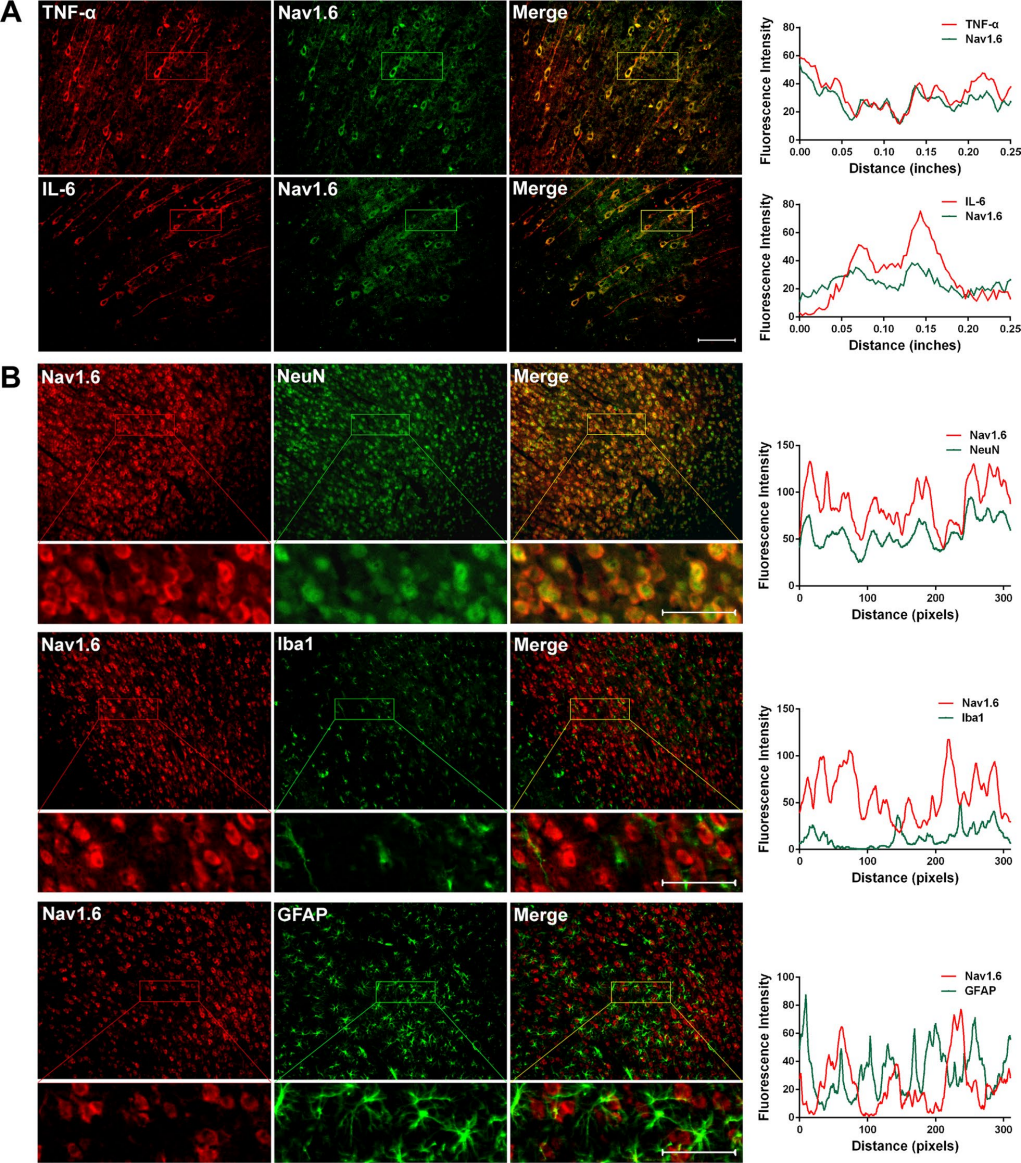
Nerve injury-induced Nav1.6 is expressed in TNF-α, IL-6-positive cells and ACC neurons. Double-immunofluorescence staining showing that **A** upregulated Nav1.6-IR (green) 7d post-SNI is co-localized with TNF-α- and IL-6-IR (red), and **B** Nav1.6-IR (red) is co-localized with NeuN (green), but not Iba1 (green) and GFAP (green) 7d post-SNI. Higher magnification micrographs from boxed areas are shown below each group. Scale bar = 50 μm. The fluorescence intensity curves for red and green from boxed areas are shown on the right side of each group

Double-immunofluorescence staining indicated that Nav1.6 colocalizes with both TNF-α and IL-6 in ACC neurons (Fig. 7A). Furthermore, Nav1.6 is believed to be the predominant sodium channel isoform in microglia [57, 58] and plays a role in microglial migration [59]. This study shows that SNI induces an increase in fluorescence intensity of microglia with an activated amoeboid shape in bilateral ACC (Additional file 3: Fig. S3). In order to establish the correlation between Nav1.6 and microglial activation, the cellular localization of Nav1.6 was examined in ACC. Dual immunolabeling showed that Nav1.6 colocalizes mainly with NeuN, but not with Iba1 or GFAP 7d after SNI (Fig. 7B), this finding indicates that there is no direct effect of Nav1.6 on glial cells.

### Chemokine CX3CL1 in ACC mediates the descending facilitation and aggravates the spinal neuroinflammation

**Fig. 8:**
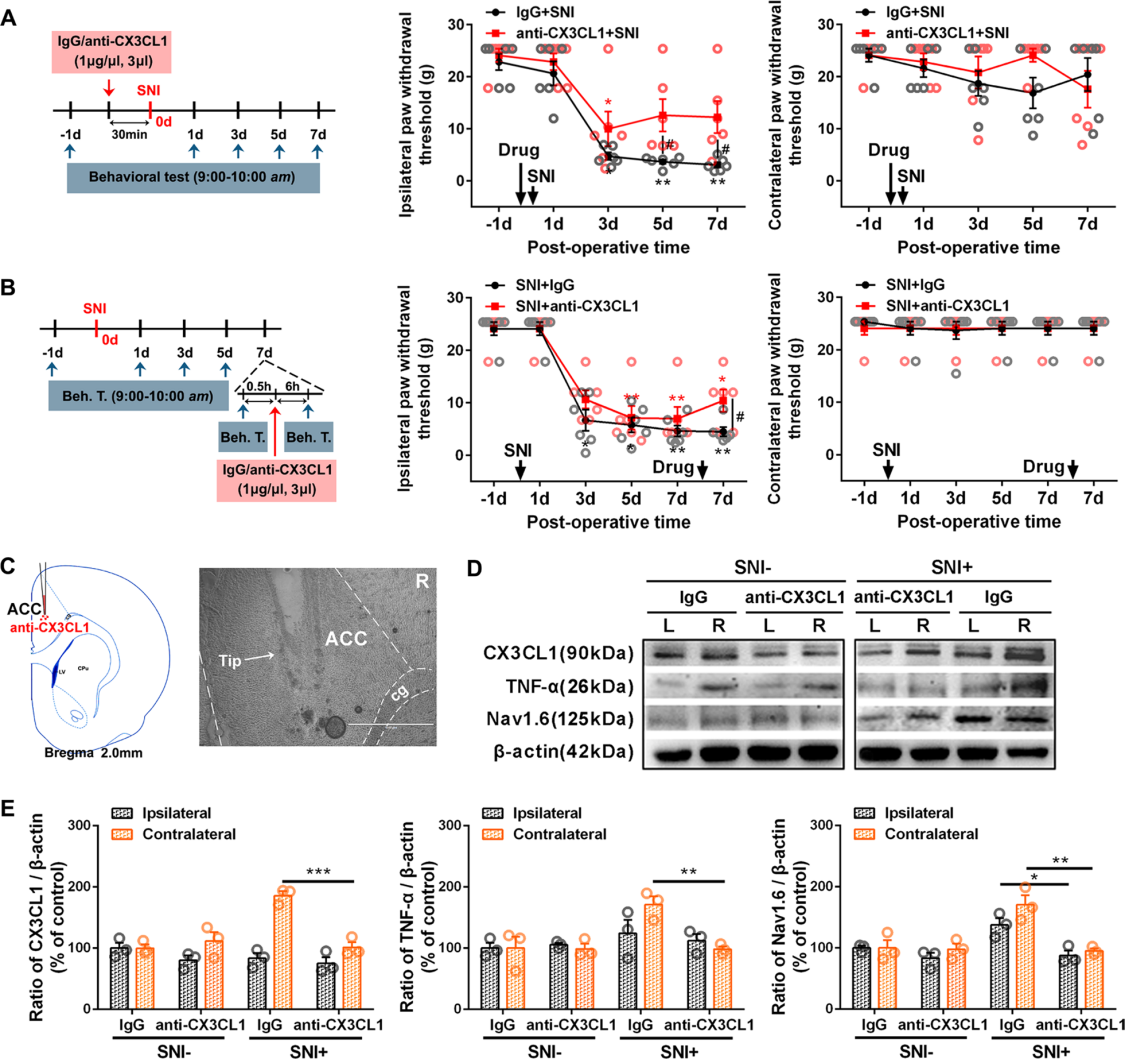
Microinjection of anti-CX3CL1 antibody into ACC attenuates SNI-induced mechanical allodynia and inhibits ACC TNF-α and Nav1.6 protein upregulations. **A**, **B** Behavioral test paradigms are shown on the first left, and the ipsilateral and contralateral test results are shown on the second and first right, respectively. Statistical significance was determined by Dunn's multiple comparisons test (*p < 0.05; **p < 0.01 versus PO -1d) or multiple t tests (#*p* < 0.05 versus IgG control). **C** The mark left by the cannula implantation showing the drug injection site in Cg1 region. **D** Representative western blotting of CX3CL1, TNF-α and Nav1.6 in bilateral ACC following contralateral administration of anti-CX3CL1 antibody (1 μg/μl, 3 μl) 7d post-SNI. **E** The quantification results showing the inhibitory effects of anti-CX3CL1 antibody on SNI-induced CX3CL1, TNF-α and Nav1.6 expressions. *p < 0.05, **p < 0.01, ***p < 0.001 versus IgG control (one-way ANOVA)

To verify the role of ACC CX3CL1 on modulating the nerve injury-associated chronic pain, the neutralization effects of CX3CL1 antibody (Fig. 8C) were tested on SNI-associated pain behaviors (Fig. 8A, B) and the expression of TNF-α and Nav1.6 in the ACC (Fig. 8D, E). As shown in Fig. 8A, anti-CX3CL1 antibody (1 μg/μl, 3 μl) or the same dose of control IgG solution were injected into the contralateral ACC 30 min before SNI surgery. Pretreatment with anti-CX3CL1 antibody but not the IgG greatly increased mechanical paw withdrawal threshold of the injury side at PO 5 and 7d (*p* < 0.05 versus IgG treatment group), suggesting that pretreatment with anti-CX3CL1 antibody effectively inhibits the induction of mechanical allodynia by SNI. Another group of behavioral tests performed on the 7th day post-SNI, at 0.5 h before and 6 h after microinjection of anti-CX3CL1 antibody (1 μg/μl, 3 μl) are shown in Fig. 8B. Anti-CX3CL1 antibody but not the IgG treatment into the contralateral ACC partially blocked the maintenance of SNI-induced allodynia, showing a higher ipsilateral paw withdrawal threshold compared to the IgG vehicle group (*p* < 0.05), but a lower threshold compared to PO-1d (*p* < 0.05). In addition, in this group, anti-CX3CL1 antibody treatment successfully reversed the upregulation of CX3CL1, TNF-α and Nav1.6 protein levels detected by western blotting in the ACC immediately dissected from the brain after behavioral tests (Fig. 8D, E).

**Fig. 9:**
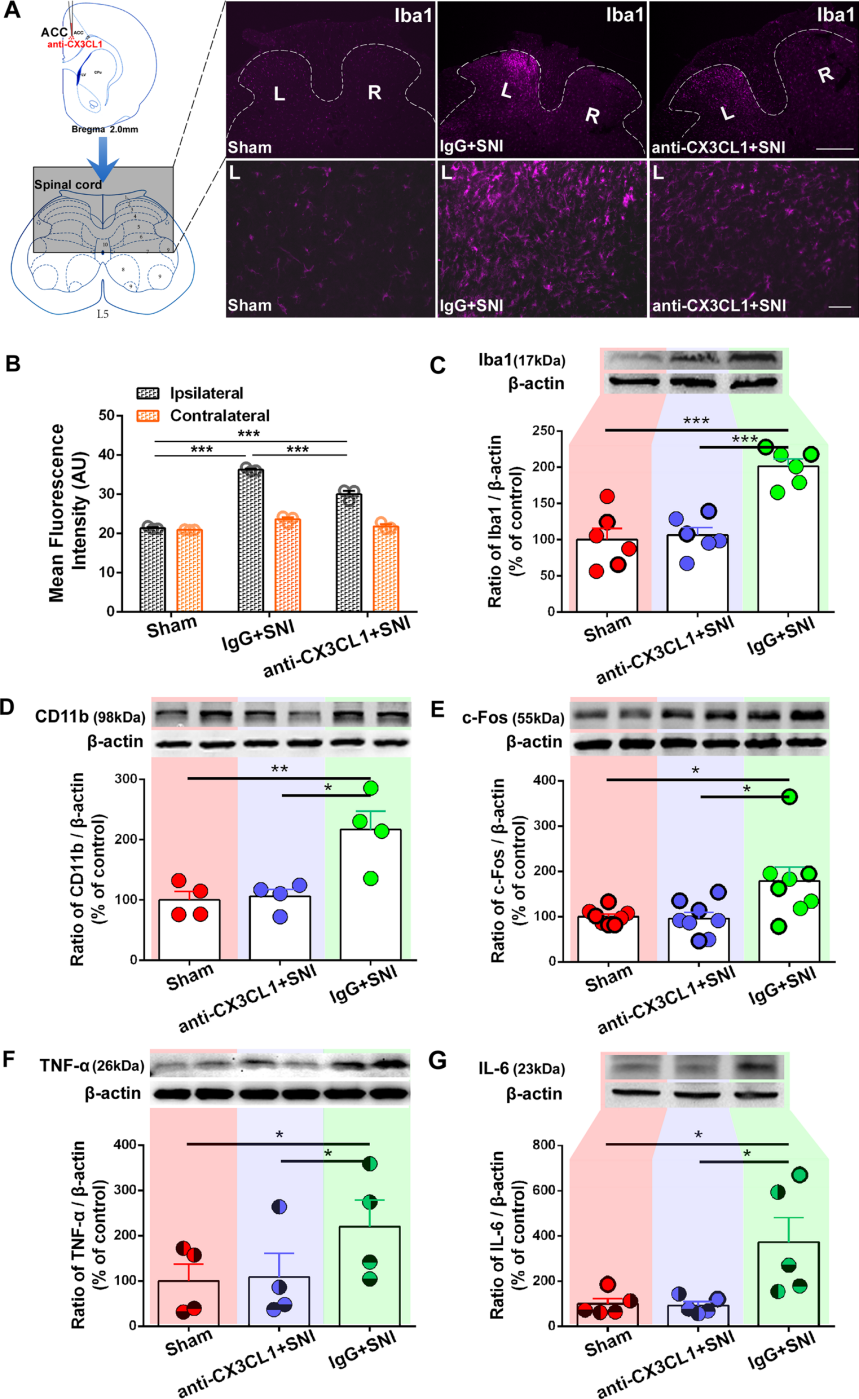
Pretreatment with anti-CX3CL1 antibody in contralateral ACC modulates the expression levels of spinal c-Fos, Iba1, TNF-α, IL-6 after SNI surgery. **A** Pretreatment with anti-CX3CL1 antibody in contralateral ACC (1 μg/μl, 3 μl) 30 min before nerve injury downregulates Iba1-IR in ipsilateral SDH 7d post-SNI. The injection site in ACC and the detection area by grey rectangular box in spinal cord are shown on the left. Representative images obtained from sham and SNI rats pretreated with IgG or anti-CX3CL1 antibody (upper right). Scale bar = 500 μm. Insets from ipsilateral SDH (lower right). Scale bar = 50 μm. **B** Quantification for mean fluorescence intensity of Iba1 from bilateral SDH (***p < 0.001, two-way ANOVA). **C**–**G** The expression levels of Iba1, CD11b, c-Fos, TNF-α and IL-6 in ipsilateral SDH following anti-CX3CL1 treatment in contralateral ACC and quantification for western blot data. *p < 0.05, **p < 0.01, ***p < 0.001 (paired t test or one-way ANOVA)

To further investigate the mechanism underlying pain modulation of CX3CL1 in ACC, the effects of contralateral ACC anti-CX3CL1 antibody on SNI-induced spinal neuroinflammation were tested (Fig. 9). The SNI-induced Iba1-IR was increased significantly in ipsilateral but not contralateral spinal dorsal horn (SDH) 7d after SNI in the IgG pretreatment group, however, it was partially inhibited in the anti-CX3CL1 antibody (1 μg/μl, 3 μl) group, showing lower fluorescence intensity compared to the IgG group (*p* < 0.001) but higher intensity compared to the sham group (*p* < 0.001, Fig. 9A, B). Western blot analysis further confirmed the inhibition of the contralateral ACC anti-CX3CL1 antibody on the ipsilateral SDH Iba1 and CD11b, microglia markers (Fig. 9C, D).

Furthermore, the contralateral ACC anti-CX3CL1 also blocked SNI-induced spinal c-Fos, TNF-α and IL-6 expressions on the ipsilateral side (Fig. 9E–G).

## Discussion

In this work, we found that elevated expressions of CX3CL1, TNF-α and IL-6 in ACC induced by unilateral nerve injury were observed on the contralateral side in the SNI group but on the bilateral side in the L5-VRT group, representing a stronger immune response to L5-VRT surgery. Both SNI and L5-VRT induced robust bilateral increase of Nav1.6 protein levels in remote ACC, but the L5-VRT-induced Nav1.6 response occurred earlier than that induced by SNI. Modulating ACC glutamatergic neurons via hM3Dq-or hM4Di-DREADD, greatly changed the levels of CX3CL1 in the ACC. Treatment with anti-CX3CL1 antibody in ACC effectively blocked the induction and the maintenance of mechanical allodynia and eliminated SNI-induced upregulations of CX3CL1, TNF-α and Nav1.6 protein levels in ACC. Contralateral ACC anti-CX3CL1 also inhibited ipsilateral spinal Iba1, c-Fos, TNF-α and IL-6 upregulations, suggesting that CX3CL1 in ACC mediates the descending facilitation and aggravates the spinal neuroinflammation.

### Stronger immune activation in ACC is responsible for mirror-image pain

Studies have shown that immune activation near intact peripheral nerves induces mechanical allodynia [4, 5] and the degree of immune activation determines whether the pain phenotype is unilateral or bilateral, i.e., the mirror-image pain requires more intense immune activation [3, 22]. Contralateral pain can be induced using SNI, an ipsilateral pain phenotype in vasoactive intestinal peptide (VIP)-deficient mice in which a stronger early proinflammatory cytokine response and a more pronounced microglial reactivity are observed in bilateral lumbar SDH compared to wild type controls [22]. Compared with SNI model [60], motor fiber injury by L5-VRT is more effective in increasing neuroimmune responses in the spinal cord [42, 61], probably because the nerve degeneration process at the site of ventral root injury produces high amounts of degradation products and induces robust release of proinflammatory mediators, which act faster and stronger on neighboring spinal cord, DRG and afferent fibers [9, 10, 21].

Spinal nociceptive transmission is under descending biphasic modulation from supraspinal structures [62]. Whether the descending net facilitation from ACC, a key factor of chronic pain, is involved in the spinal immune activation and thus contributes to mirror pain remains unclear. It has been reported that cytokines can cross the blood–brain barrier by saturable transport systems [63, 64]. Increased TNF-α in cerebrospinal fluid and in the plasma is observed after SNI [65]. However, our recent work showed that SNI elevated TNF-α levels in contralateral but not ipsilateral ACC which contributed to ACC hyperexcitability, pain aversiveness and pain maintenance [33], suggesting a predominant effect of spinal nociceptive input on ACC TNF-α expression. Furthermore, modulating ACC glutamatergic neurons via chemogenetic technology DREADD (designer receptors exclusively activated by designer drugs) greatly changes ACC TNF-α levels and mechanical paw withdrawal threshold [33]. The positive interactions between TNF-α and ACC neurons might further enhance the descending facilitation to spinal cord, participating in the maintenance of neuropathic pain. By comparing the spatiotemporal variations of TNF-α and IL-6 levels in ACC between SNI and L5-VRT rats, this study indicates that L5-VRT induces a stronger immune activation in ACC manifested by enhanced TNF-α and IL-6 expression on both sides of ACC (Fig. 1) and early bilateral upregulation of their upstream chemokine CX3CL1 (Fig. 3). Therefore, we speculated that the stronger neuroinflammation in ACC enhanced spinal response to nociceptive transmission, which may contribute to the formation of mirror pain.

### Chemokine CX3CL1 in ACC mediates the descending facilitation and aggravates the spinal neuroinflammation

The chemokines upregulated in the spinal cord after peripheral nerve injury and involved in the development of chronic pain were recently reviewed [46]. However, the role of chemokines in supraspinal ACC in pain processing has rarely been reported. Recent reports indicate that the C-X-C motif chemokine 13 (CXCL13)/CXCR5 signaling in the ACC is involved in neuropathic pain-related aversion but not in spinal nerve ligation (SNL)-induced mechanical allodynia [66]. Furthermore, C-X-C motif receptor 3 (CXCR3) contributes to hyperalgesia following chronic constriction injury (CCI) of the sciatic nerve [67]. Based on data from protein microarray, this study focuses on the role of CX3CL1 in ACC nociceptive processing. CX3CL1 (also called fractalkine), the only member of the C-X3-C group, is constitutively expressed in the neurons of the normal peripheral nervous system and the central nervous system [68, 69]. Chemokine pairs CX3CL1/CX3CR1 are involved in neuropathic pain via neuron-microglia interaction in the spinal cord [70–72]. P38, a member of mitogen-activated protein kinases (MAPKs), is an important downstream kinase of CX3CL1/CX3CR1 signaling [73–75]. Activation of CX3CL1/CX3CR1/p38 MAPK pathway induces synthesis of proinflammatory cytokines such as TNF-α and IL-6 in the microglia [75, 76].

**Fig. 10:**
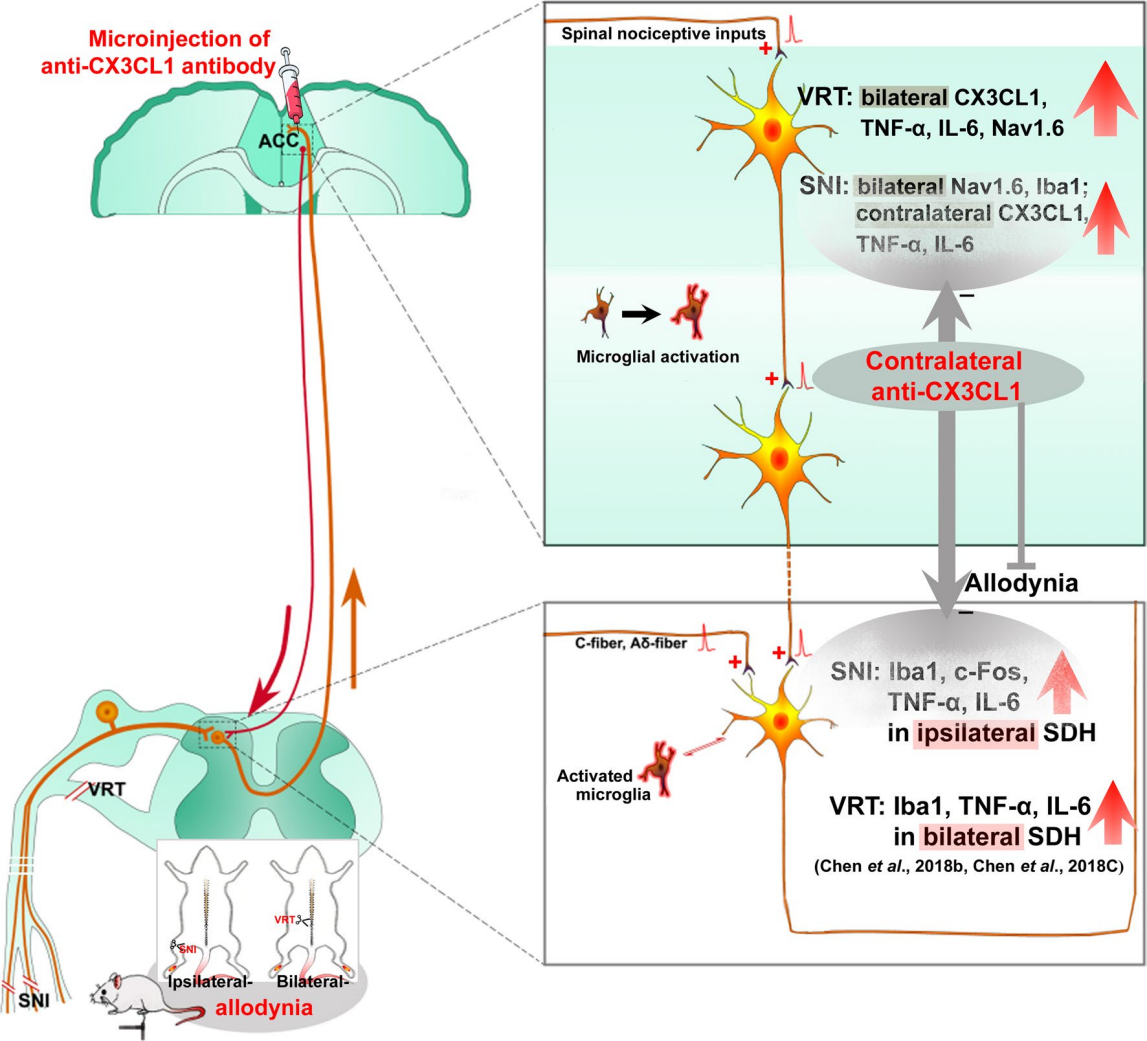
Graphical abstract: anterior cingulate cortex (ACC), a first-order cortical region that responds to painful stimuli, may play important roles in the occurrence of mirror-image pain. In this study, we provide evidence that in ACC, stronger immune response to L5-VRT surgery contributes to mirror pain, and CX3CL1 mediates the descending facilitation and aggravates the spinal neuroinflammation. Strategies targeting chemokine-mediated ACC hyperexcitability may develop novel therapeutics for neuropathic pain

Studies have shown that noxious information from either the limb or the face can ascend ipsilaterally to the thalamus in humans as well as in animals in addition to the traditional contralateral ascending pathway [77, 78]. These studies are consistent with our recent work that the IR of c-Fos, a widely used activity marker [79], increases in the ACC 1 h post-SNI and to greater and lesser extents in contralateral and ipsilateral sides, respectively [33]. As mentioned above, the projection of thalamic MD nucleus to ACC governs endogenous descending facilitation in pain regulation [24, 80]. Xiao et al. demonstrated that lesion of the contralateral but not ipsilateral thalamic MD nucleus inhibits bilateral Fos expression within the cingulate cortex during hypertonic saline-induced muscle nociception [25], a model for mirror pain [81]. This suggests that the excitation in bilateral cerebral cortices is not dependent on the projections from the spinal cord but on the cross-talk of neuronal activities within cerebral cortices via transcallosal pathways [25, 82, 83]. In this study, we found that L5-VRT-induced CX3CL1 expression to be almost equally upregulated at an early stage (within 15 h) in bilateral ACC, with the contralateral prior to the ipsilateral (Fig. 3). Modulating ACC glutamatergic neurons in SNI mice by hM4Di or in normal mice by hM3Dq, greatly changed the ACC CX3CL1 levels and the mechanical paw withdrawal threshold (Fig. 5). We cannot exclude the impact of bilateral spinal cord on early activity of ACC after nerve injury, because the upregulations of TNF-α and IL-6 as well as the activation of microglia in bilateral SDH and ventral horn induced by L5-VRT appear earlier postoperatively, within half an hour on the ipsilateral side and slightly delayed on the contralateral side [9, 10]. In other words, the early neuroinflammation of bilateral spinal cord caused by L5-VRT and its secondary excitatory afferent may ascend to ACC, increasing its activity and upregulating CX3CL1 (Fig. 10).

In addition, microinjection of CX3CL1 neutralizing antibody (1 μg/μl, 3 μl) into the contralateral ACC downregulated Iba1, c-Fos, TNF-α, and IL-6 in ipsilateral SDH induced by SNI (Fig. 9), suggesting that the top-down descending facilitation from ACC to spinal cord mediated by CX3CL1 existed and enhanced the spinal immune activation. Obviously, it is not difficult to infer that upregulation of CX3CL1 in bilateral ACC following L5-VRT can facilitate the response of bilateral spinal cord to nociception. Interaction between spinal cord and ACC activity contributes to the formation of bilateral pain induced by unilateral nerve injury. The dysregulation of CX3CL1 in the bilateral ACC after motor nerve injury may be also derived from the intercortical communication from contra- to ipsilateral ACC [25] or stronger peripheral inflammation through the damaged blood–brain barrier input [63, 64, 84, 85].

### CX3CL1/TNF-α/Nav1.6 pathway is responsible for ACC hyperexcitation

ACC neurons are prone to nerve injury-induced synaptic modifications and to increased spontaneous membrane-potential oscillations [86–88]. Synaptic plasticity induced in ACC by glutamatergic neurons of layers II/III and V is causally related to chronic pain [37]. Pyramidal cells in ACC layer V directly [28] or indirectly [89] project to the SDH and are further involved in the descending modulation of spinal sensory transmission. Spontaneous firing occurs in ACC layers II/III and V. Such firings are initiated by thalamocortical synaptic inputs from the thalamic MD nucleus and are maintained by intracortical neuronal depolarized upstate mechanisms in physiological and pathological pain states [90, 91]. Pharmacological inhibition of the spontaneous firing in ACC increases the rat paw withdrawal threshold and reduces pain-related anxiety-like behavior [91]. However, the underlying molecular mechanism is unclear.

The voltage-gated sodium channel isoform Nav1.6 can relay excitatory persistent and resurgent currents in DRG and cerebral cortex [92–96]. Both currents play a role in the production of spontaneous activities such as the persistent current by providing a depolarizing contribution to membrane-potential oscillations, and the resurgent current by allowing high frequency repetitive firing [93, 97]. In DRG, spontaneously active bursting cells express high levels of Nav1.6 and knockdown of Nav1.6 completely blocks the abnormal spontaneous activity as well as pain behaviors [53, 54, 98–100]. Although it has been reported that the mRNA expression of Nav1.6 is upregulated in the ACC during paclitaxel-induced neuropathic pain [101], the role of Nav1.6 in ACC following peripheral nerve injury is largely unknown. Another reason why we chose to study the role of Nav1.6 in pain processing in ACC is that TNF-α can directly regulate its expression and function. TNF-α epigenetically upregulates Nav1.6 expression via activating the signal transducer and activator of transcription-3 (STAT3) pathway and also promotes the trafficking of Nav1.6 to the membrane of DRG neurons, an essential step in mediating neuronal excitability and repetitive firing, thus contributes to neuropathic pain induced by L5-VRT [100, 102].

This study reveals that peripheral nerve injury induces robust increase in Nav1.6 protein levels in remote ACC neurons. Although both SNI and L5-VRT-induced upregulation of Nav1.6 was bilateral in ACC, the L5-VRT-induced Nav1.6 response appeared earlier, 3 days after surgery (Fig. 6). Microinjection of anti-CX3CL1 antibody inhibits the abnormal expression of TNF-α and Nav1.6 in ACC while relieving mechanical allodynia (Fig. 8), suggesting stronger activities of bilateral cerebral cortices may contribute predominantly to the occurrence and development of mirror or contralateral pain. What is puzzling is that in our previous work [33] and this study, SNI induced the expression of CX3CL1, TNF-α and IL-6 in ACC on the contralateral side of the nerve injury, while the abnormal expression of Nav1.6 was on the bilateral side. There must be other mechanisms besides TNF-α to regulate the expression of Nav1.6 in bilateral ACC. CX3CL1 expressed in neurons can induce microglial activation via its microglial receptor CX3CR1 (neuron-to-microglia signaling) [103, 104]. The data in this study indicate that SNI-induced IL-6 expression in ACC neurons as well as in microglial cells. The complex network architecture in ACC [37, 105] as well as the cross-talk between ACC neurons and microglia might be involved in the bilateral abnormal expression of Nav1.6. Interestingly, in addition to regulating the excitability of neurons, sodium channel activity modulates multiple functions in microglia such as phagocytosis, cytokine release and migration [59]. In addition the increase in VGSC inward current elicits microglial activation [58]. Nav1.6 is expressed in primary microglia after spinal cord injury [58] and its activity contributes to the response of microglia to multiple activating signals [57]. In this work, we found that SNI induced microglial activation with an activated amoeboid shape in bilateral ACC (Additional file 3: Fig. S3), but Nav1.6 did not colocalize with Iba1-positive cells (Fig. 7), negating potential involvement of Nav1.6 in this activation. The activation mechanism of microglia in ACC after nerve injury and the role of microglia in pain information processing remain to be further studied.

## Conclusions

It is now clear that the descending pain modulatory pathway can be both facilitatory and inhibitory, with a dynamic balance between the two functions [62]. When acute pain turns to chronic pain, the descending facilitation function mediated by CX3CL1 and its downstream cascade may play a pivotal role, leading to enhanced pain sensitization and even mirror-image pain. Strategies that target chemokine-mediated ACC hyperexcitability may lead to novel therapies for the treatment of neuropathic pain.

## Supplementary Information

**Additional file 1: Fig. S1**. Determination of specificity of CaMKII-positive neurons transfected with DREADD-hM3Dq and hM4Di virus. (A) Schematic diagram of the time points for virus injection carrying hM3Dq-, hM4Di-mCherry or mCherry in contralateral ACC and SNI surgery. (B) Double-immunofluorescence staining shows the co-localization of mCherry (red), hM3Dq-mCherry (red), hM4Di-mCherry (red) and CaMKII (green). (C) The percentage of virus-transfected neurons that are CaMKII-positive is shown. *p < 0.05 versus mCherry control groups (*n* = 4 mice/group, one-way ANOVA). Scale bar = 50 μm.

**Additional file 2: Fig. S2**. The quantity difference of co-labeled mCherry and c-Fos in different virus transfection groups following administration of CNO. (A) Schematic diagram of the time points for virus injection carrying hM3Dq-, hM4Di-mCherry or mCherry in contralateral ACC and SNI surgery. (B) Double-immunofluorescence staining shows an increased co-labeled percentage of hM3Dq-mCherry-IR (red) and c-Fos-IR (green) and a decreased percentage of hM4Di-mCherry-IR (red) and c-Fos-IR (green) co-localized (yellow) in all transfected neurons (red) following CNO (5.0 mg/kg, i.p.). (C) The percentage of co-localization is shown. *p < 0.05 versus mCherry control groups (*n* = 4 mice/group, one-way ANOVA). Scale bar = 50 μm.

**Additional file 3: Fig. S3**. Increased fluorescence intensity of microglia with an activated amoeboid shape observed in bilateral ACC 7d post-SNI. (A) Representative results of Iba1 in bilateral ACC observed in naive and SNI rats are shown. Scale bar = 50 μm. (B) Quantification for mean fluorescence intensity of Iba1 in ipsilateral and contralateral sides. ***p < 0.001 versus naive group (one-way ANOVA).

## Data Availability

All the necessary data are included within the article. Further data will be shared by request.

## Abbreviations

ACC: Anterior cingulate cortex
SNI: Spared nerve injury
L5-VRT: L5 ventral root transection
DREADD: Designer receptors exclusively activated by designer drugs
TNF-α: Tumor necrosis factor alpha
IL-6: Interleukin-6
PO: Postoperative
VGSC: Voltage-gated sodium channel
NP: Neuropathic pain
CSF: Cerebrospinal fluid
DRG: Dorsal root ganglia
MD: Mediodorsal
PB: Phosphate buffer
FITC: Fluorescein isothiocyanate
IR: Immunoreactivity
hM4Di: Gi-coupled human M4 muscarinic receptor
hM3Dq: Gq-coupled human M3 muscarinic receptor
AP: Anteroposterior
ML: Mediolateral
DV: Dorsoventral
CNO: Clozapine-*N*-oxide
DMSO: Dimethylsulfoxide
SDH: Spinal dorsal horn
VIP: Vasoactive intestinal peptide
CXCL13: C-X-C motif chemokine 13
CXCR3: C-X-C motif receptor 3
MAPKs: Mitogen-activated protein kinases
STAT3: Signal transducer and activator of transcription-3
