# Supplementary figures and images for: Neuroinflammation in the anterior cingulate cortex: the potential supraspinal mechanism underlying the mirror-image pain following motor fiber injury
Source: J Neuroinflammation. 2022 Jun 20;19:162. doi: 10.1186/s12974-022-02525-8 (PMC9210588; doi:10.1186/s12974-022-02525-8)

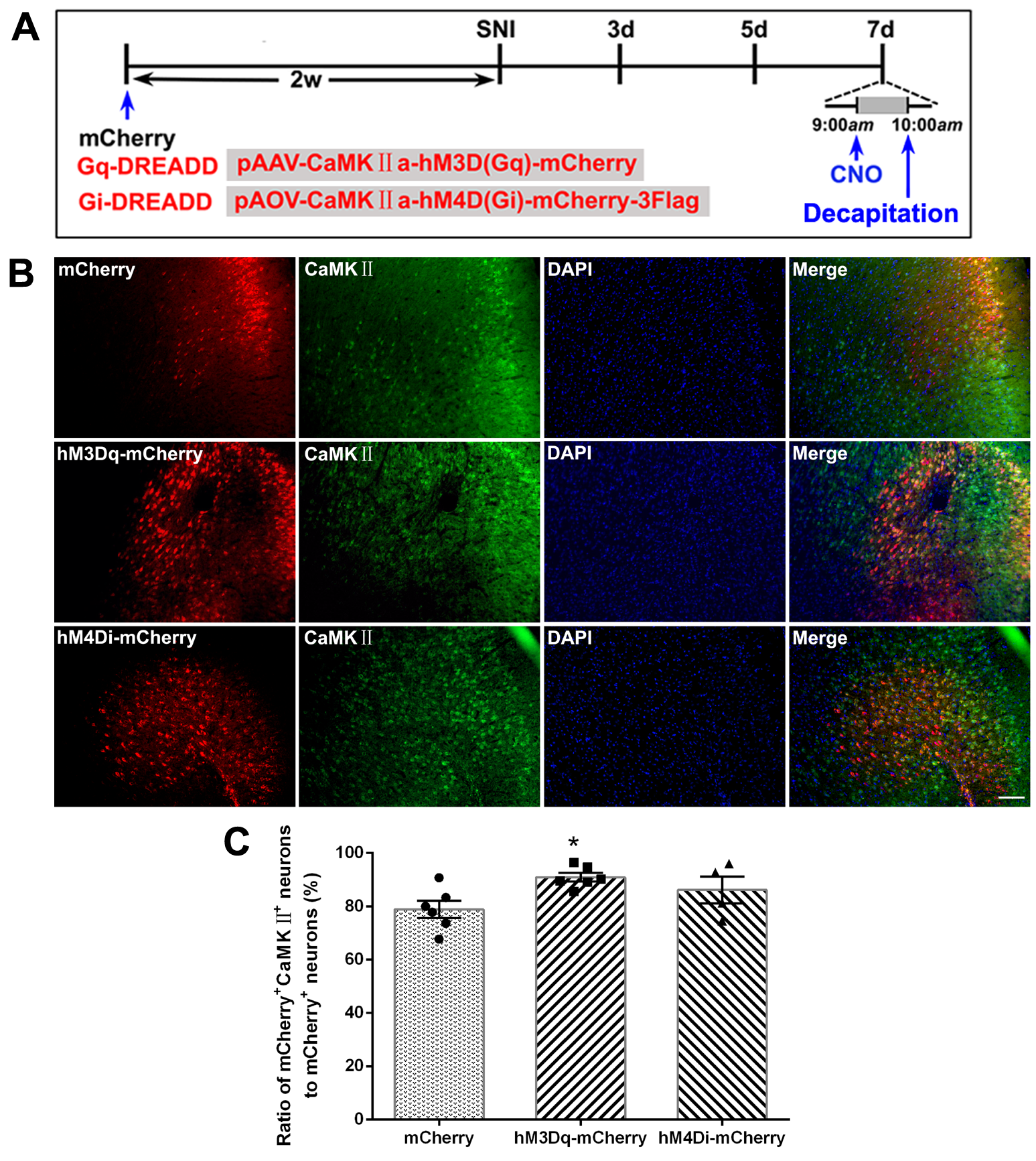

Supplement: Supplementary file 1 — Additional file 1: Fig. S1. Determination of specificity of CaMKII-positive neurons transfected with DREADD-hM3Dq and hM4Di virus. (A) Schematic diagram of the time points for virus injection carrying hM3Dq-, hM4Di-mCherry or mCherry in contralateral ACC and SNI surgery. (B) Double-immunofluorescence staining shows the co-localization of mCherry (red), hM3Dq-mCherry (red), hM4Di-mCherry (red) and CaMKII (green). (C) The percentage of virus-transfected neurons that are CaMKII-positive is shown. *p < 0.05 versus mCherry control groups (n = 4 mice/group, one-way ANOVA). Scale bar = 50 μm. [file 12974_2022_2525_MOESM1_ESM.tif]

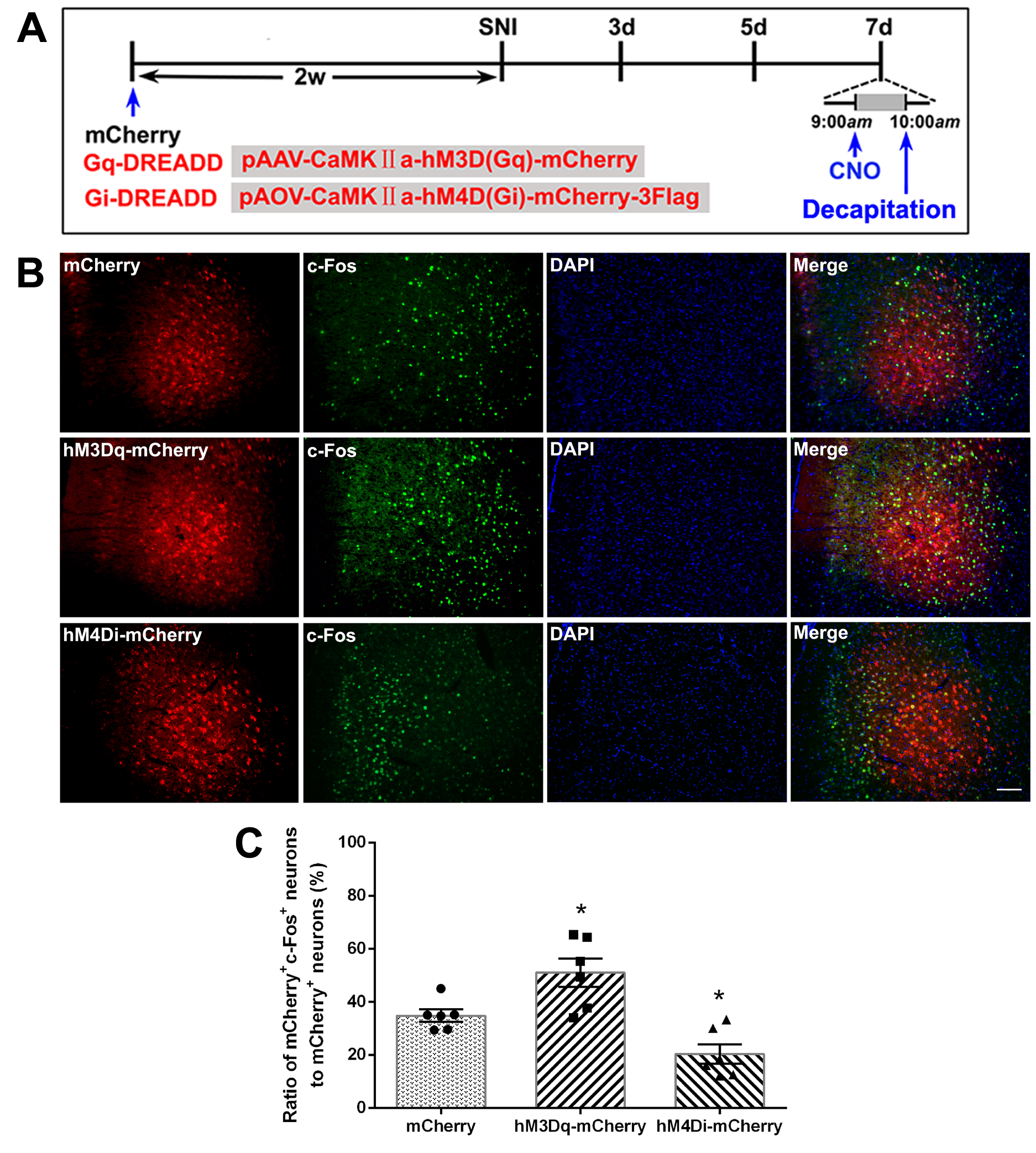

Supplement: Supplementary file 2 — Additional file 2: Fig. S2. The quantity difference of co-labeled mCherry and c-Fos in different virus transfection groups following administration of CNO. (A) Schematic diagram of the time points for virus injection carrying hM3Dq-, hM4Di-mCherry or mCherry in contralateral ACC and SNI surgery. (B) Double-immunofluorescence staining shows an increased co-labeled percentage of hM3Dq-mCherry-IR (red) and c-Fos-IR (green) and a decreased percentage of hM4Di-mCherry-IR (red) and c-Fos-IR (green) co-localized (yellow) in all transfected neurons (red) following CNO (5.0 mg/kg, i.p.). (C) The percentage of co-localization is shown. *p < 0.05 versus mCherry control groups (n = 4 mice/group, one-way ANOVA). Scale bar = 50 μm. [file 12974_2022_2525_MOESM2_ESM.tif]

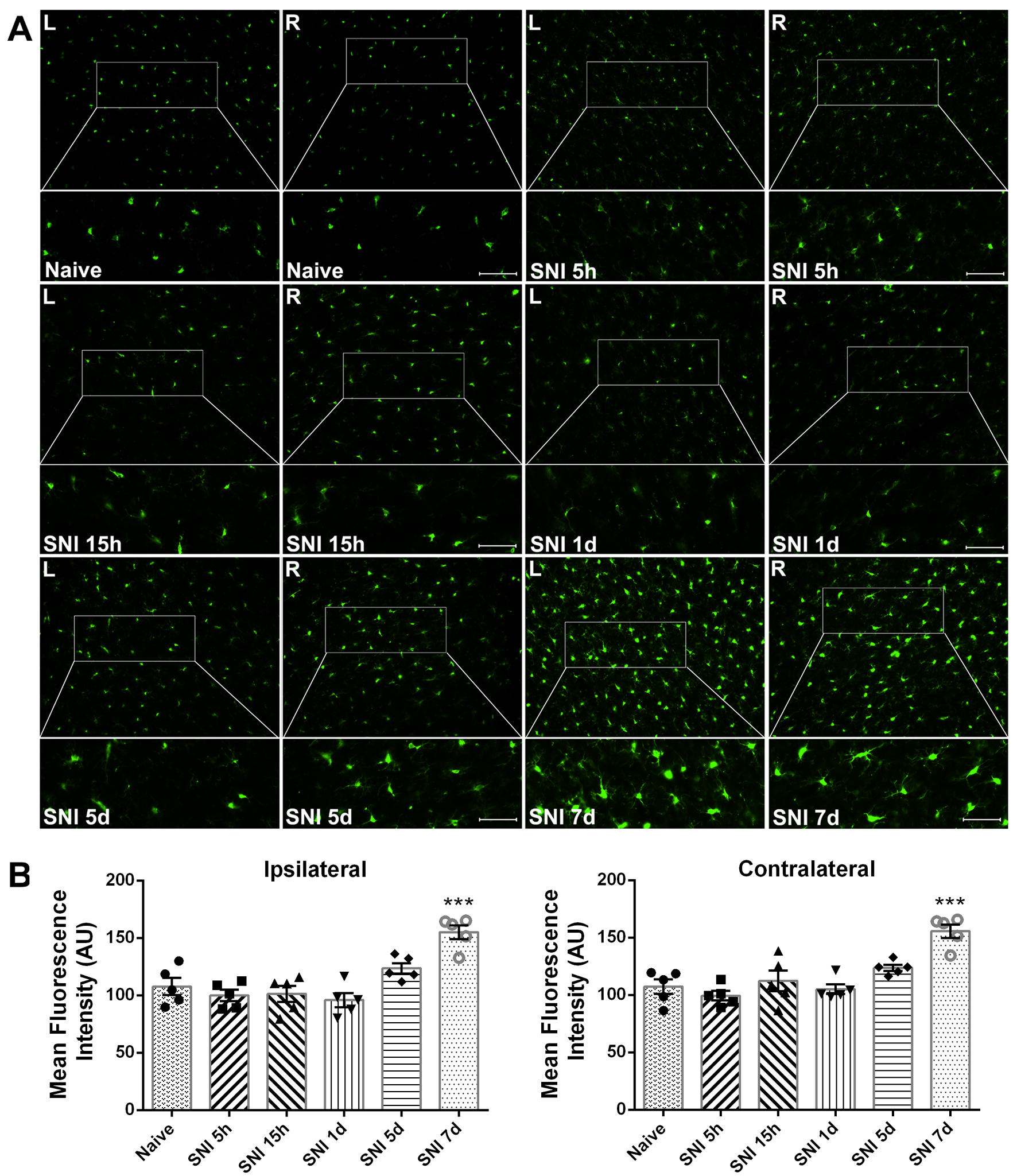

Supplement: Supplementary file 3 — Additional file 3: Fig. S3. Increased fluorescence intensity of microglia with an activated amoeboid shape observed in bilateral ACC 7d post-SNI. (A) Representative results of Iba1 in bilateral ACC observed in naive and SNI rats are shown. Scale bar = 50 μm. (B) Quantification for mean fluorescence intensity of Iba1 in ipsilateral and contralateral sides. ***p < 0.001 versus naive group (one-way ANOVA). [file 12974_2022_2525_MOESM3_ESM.tif]
